# Evaluation of a deep learning magnetic resonance imaging reconstruction method for synthetic computed tomography generation in prostate radiotherapy

**DOI:** 10.1016/j.phro.2024.100557

**Published:** 2024-02-16

**Authors:** Lars E. Olsson, Sacha af Wetterstedt, Jonas Scherman, Adalsteinn Gunnlaugsson, Emilia Persson, Christian Jamtheim Gustafsson

**Affiliations:** a Radiation Physics, Department of Hematology, Oncology, and Radiation Physics, Skåne University Hospital, Klinikgatan 5, Lund 221 85, Sweden; b Department of Translational Medicine, Medical Radiation Physics, Lund University, Carl Bertil Laurells gata 9, Malmö 205 02, Sweden

**Keywords:** Synthetic CT, Radiotherapy, Prostate cancer, MRI, Deep learning reconstruction

## Abstract

**Background and Purpose:**

In magnetic resonance imaging (MRI) only radiotherapy computed tomography (CT) is excluded. The method relies entirely on synthetic CT images generated from MRI. This study evaluates the compatibility of a commercial synthetic CT (sCT) with an accelerated commercial deep learning reconstruction (DLR) in MRI-only prostate radiotherapy.

**Materials and Methods:**

For a group of 24 patients (cohort 1) the effects of DLR were studied in isolation. MRI data were reconstructed conventionally and with DLR from identical k-space data, and sCTs were generated for both reconstructions. The sCT quality, Hounsfield Unit (HU) and dosimetric impact were investigated. In another group of 15 patients (cohort 2) effects on sCT generation using accelerated MRI acquisition (40 % time reduction) reconstructed with DLR were investigated.

**Results:**

sCT images from both cohorts, generated from DLR MRI data, were of clinically expected image quality. The mean dose differences for targets and organs at risks in cohort 1 were <0.06 Gy, corresponding to a 0.1 % prescribed dose difference. Similar dose differences were observed in cohort 2. Gamma pass rates for cohort 1 were 100 % for criteria 3 %/3mm, 2 %/2mm and 1 %/1mm for all dose levels. Mean error and mean absolute error inside the body, between sCTs, averaged over all cohort 1 subjects, were −1.1 ± 0.6 [−2.4 0.2] and 2.9 ± 0.4 [2.3 3.9] HU, respectively.

**Conclusions:**

DLR was suitable for sCT generation with clinically negligible differences in HU and calculated dose compared to the conventional MRI reconstruction method. For sCT generation DLR enables scan time reduction, without compromised sCT quality.

## Highlights

• Demonstration showed feasibility of combining commercial deep learning for MRI reconstruction and synthetic CT generation.
• Differences in synthetic CT generated with and without deep learning MRI reconstruction were clinically negligible.
• Commercial deep learning-based MRI reconstruction reduced scan time with 40%.

## 1 Introduction

One of the recent developments in radiation therapy (RT) is magnetic resonance imaging (MRI)-Only RT treatment planning. The rationale is to exclude the computed tomography (CT) examination and thereby simplify the workflow and avoid image registration uncertainties between CT and MRI [1], [2]. The electron density maps required by the treatment planning system for dose calculation are provided by synthetic CT (sCT) images generated from MR images [3]. MRI is a slow imaging technique compared to CT, and the MR images are prone to low signal-to-noise ratio (SNR) and motion artifacts. Recently, deep learning-based MRI reconstruction (DLR) has been introduced, with considerable noise reduction and without loss of image quality. The improved SNR can be traded for either improved image resolution or reduced MRI acquisition time. For example, in diagnostic radiology it has been possible to reduce the in-vivo scan time with 50 % for T2-weighted (T2w) brain imaging [4]. DLR has also been proven successful for prostate and breast imaging by providing substantial noise reduction without any noticeable image blurring or loss of resolution in T2w images [5], [6].

The requirements on MR images for use in RT differ in many aspects compared to diagnostic radiology. In RT there is a higher requirement on spatial accuracy, signal stability and image homogeneity and this is especially relevant for MR images used for generation of sCT [7], [8], [9]. Therefore, it would be of interest to apply DLR to MRI data for generation of sCT images of the pelvis, for which a high SNR is desirable, but the acquisition time needs to be limited to keep the effects of physiologic motion in the images on an acceptable level. DLR may be a technique that can improve the MRI-only workflow by reducing the acquisition time without any counteracting factors. In addition, many of the sCT generation products are based on deep learning methods, which are known to be sensitive to the conformity of the input data relative to the training data [10], [11]. This aspect is of particular interest to study for the sCT generation method in use.

The aims of this study were twofold: 1) to evaluate the compatibility between a commercial DLR product and a commercial sCT generation software, and 2) to validate that the quality of the sCTs remained the same when using a DLR accelerated MRI acquisition protocol for RT. The evaluation was performed on clinical MRI-only based treatment plans for prostate cancer, by quantitatively assessing the Hounsfield Units (HU) and dosimetric integrity of sCTs created from such MR images.

## 2 Material and methods

### 2.1 Patient data

Two patient cohorts with prostate cancer patients, treated with ultra-hypofractionated RT using a clinical MRI-only treatment workflow [12], were used in this study. Cohort 1 consisted of 24 prostate cancer patients and aimed to investigate the isolated effect of a commercial DLR method on MRI images with respect to sCT generation. Specifically, the GE HealthCare AIR Recon DL (GE Healthcare, Chicago, USA) method was investigated. Cohort 2 consisted of 15 prostate cancer patients and aimed to investigate the effect of the DLR method on sCT generation when Air Recon DL was used to enable an accelerated MRI acquisition protocol.

A fractionation scheme of 42.7 Gy in 7 fractions was planned (Eclipse v.15.6, Varian Medical Systems, Palo Alto, USA) and delivered for all patients using single or dual arc volumetric modulated arc therapy (VMAT) technique with a 6 MV flattening filter free energy on Varian TrueBeam linear accelerators (v2.7, Varian Medical Systems) equipped with the Millenium multileaf collimator.

The study was approved by the regional ethics board “Regionala Etikprövningsnämnden in Lund”, diary number 2013/742. No patient data used in this study is openly available.

### 2.2 Data acquisition

**Fig. 1:**
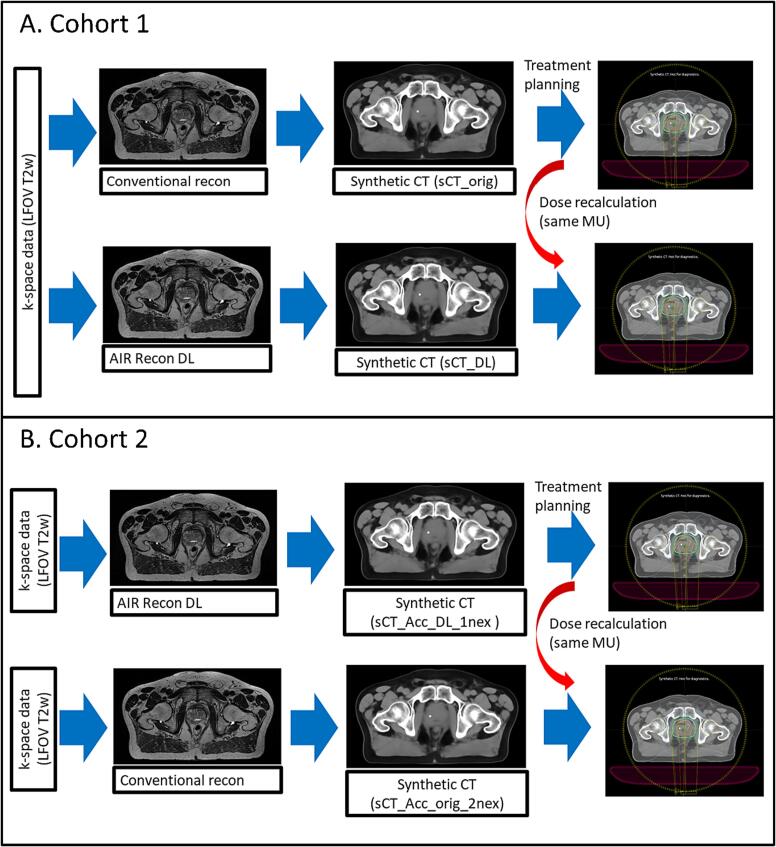
A. Data workflow for the patients in cohort 1 using two parallel image reconstruction pipelines on the same k-space data, one with conventional reconstruction (top row in A) and one with the GE HealthCare AIR Recon DL reconstruction (bottom row in A). B. Data workflow for the patients in cohort 2 using two image reconstruction pipelines, each one with unique k-space data, one with GE HealthCare AIR Recon DL reconstruction (clinical protocol, one average acquired, top row in B) and one with the conventional reconstruction (two averages acquired, bottom row in B). Images presented in B are symbolic representations and might not represent actual patient data.

Patients in cohort 1 were scanned on a GE Architect 3T (v.29.1, GE Healthcare, Chicago, USA) using a 16 channel GE HealthCare AIR Coil without any coil bridges [13]. The raw k-space data from the acquisition of a single large field-of-view (LFOV) T2w scan was used to reconstruct the MR images with the conventional vendor MR image reconstruction method and the GE HealthCare AIR Recon DL method (Air Recon DL mode set to High) (Fig. 1A). Both reconstruction methods are commercially available. Examples of T2w images for the two reconstruction methods are provided in Fig. S2 in Supplementary material.

The MRI acquisition sequence for the LFOV T2w scan in cohort 1 was a transversal 2D fast recovery fast spin echo (FRFSE) with following parameters [12]: TR 15000 ms, TE 96 ms, FOV 448 × 314 mm, acquisition matrix 640 × 512, reconstructed matrix 1024 × 1024, slice thickness 2.5 mm, slice spacing 0 mm, 88 slices, phase encoding direction anterior/posterior, number of averages was 1, no acceleration, receiver bandwidth 391 Hz/pixel, refocus flip angle 130 degrees, echo train length 15, intensity correction SCENIC, 3D distortion correction on and total acquisition time of 7:00 min.

Patients in cohort 2 were scanned on the same MRI system as cohort 1, using the same coil setup, however the system software was v.30.0. In this cohort the protocol for the LFOV T2w scan was based on the same sequence type (FRFSE) as in cohort 1. To enable undersampling and acceleration with a 40 % time reduction, the protocol was revised in its acquisition parameters compared to cohort 1 and utilized AIR Recon DL as standard image reconstruction (Air Recon DL mode set to High). This was also the default clinical sequence used for sCT generation and the acquisition parameters were: TR 13 600 ms, TE 96 ms, FOV 320 × 480 mm, acquisition matrix 384 × 360, reconstructed matrix 512 × 512, slice thickness 2.5 mm, slice spacing 0 mm, 88 slices, phase encoding direction left–right, number of averages was 1, parallel imaging acceleration factor 2.5, receiver bandwidth 434 Hz/pixel, refocus flip angle 130 degrees, echo train length 15, intensity correction SCENIC, 3D distortion correction on, and total acquisition time of 3:51 min. Screenshot of full vendor specific protocol for cohort 2 can be found in Supplementary material (Fig. S1) where image examples can be found (Fig. S3). Additionally, another separate MRI acquisition for a second LFOV T2w scan was added to this cohort in the same scan session. The acquisition and reconstruction parameters seen in Fig . S1 in Supplementary material were the same as the accelerated clinical protocol in cohort 2 except that it used the conventional vendor-based image reconstruction and had the number of averages in the acquisition set to two, instead of one (double scan time, 7:29 min). Image examples can be found in Supplementary material (Fig. S4). The number of averages of two in the second scan was set to make sure that images in the two scans had comparable SNR. Examples of T2w images for the two scans are provided in Fig. S3 and Fig. S4 in Supplementary material.

### 2.3 Deep learning-based MRI reconstruction

The AIR Recon DL method uses raw complex k-space data as pipeline input to reconstruct MR images. It can replace conventional image filters, and it instead employs a deep convolutional neural network (CNN) to adjust MR image noise, minimize truncation artifacts (Gibbs ringing) and enhance edge sharpness [4]. The CNN was trained via supervised learning on 4 million image pairs, contrasting near-perfect quality images with synthesized, lower-resolution versions featuring intentional artifacts and noise. This diverse set, spanning various anatomical structures, was further enriched with augmentations like rotations, flips, and noise for enhanced adaptability and robustness. A vendor-based description of the product is available as a white paper [14].

### 2.4 Synthetic CT generation

The reconstructed T2w images from each reconstruction method in cohort 1 were converted to sCTs using Spectronic MRI Planner (v.2.4.14, Spectronic Medical AB, Helsingborg, Sweden) [12], [15], resulting in two sCT volumes sCT_orig and sCT_DL (Fig. 1A). All sCTs had three gold fiducial markers burnt in as spherical structures with high HU in the prostate. This was performed in the MRI Planner software based on the manually identified fiducial center of mass in the T2w image. MRI Planner is based on the transfer function estimation algorithm to calculate a CT representation for an incoming MRI by first estimating the spatially variant coefficients of an affine transfer function and then applying this transfer function to the MRI. The coefficients of the transfer function are generated using a structure of deep CNN [16]. sCT generation for data in cohort 2 was performed in the same manner as described above and resulted in two sCT volumes sCT_Acc_DL_1nex and sCT_Acc_orig_2nex (Fig. 1B). Examples of sCT images from both MRI reconstruction methods in cohort 1 and 2 are provided in Fig. S5 and Fig. S6 respectively in Supplementary material.

### 2.5 Data analysis

In cohort 1, the sCT_DL was compared to sCT_orig with respect to sCT bone and soft tissue generation by calculating the difference image through image subtraction, and the volume of the body structure for both sCTs was measured. Associated T2w volumes were subtracted in the same way as the sCTs and visually compared. To assess differences between sCT_orig and sCT_DL in cohort 1, HU differences inside the patient contour (HU range −990 to 10^13), outside the patient contour (HU range −10^13 to −200), in fat (−200 to −28.5), in muscle (−28.5 to 100), in spongy bone (100 to 575) and in compact bone (575 to 10^13) were calculated on a voxel level and analyzed using MICE Toolkit (v.2021.1.0, NONPI Medical AB, Umeå, Sweden). The HU ranges were chosen according to Maspero et al [17] and mean absolute error (MAE) were calculated within each range.

To calculate the total mean dose differences for cohort 1 in planning target volume (PTV), clinical target volume (CTV) and organs at risk, the clinical RT structures, except the body structure, were copied from the sCT_orig to the sCT_DL. The clinical treatment plans, created using sCT_orig, were copied to sCT_DL and the dose was recalculated with the Anisotropic Analytical Algorithm (AAA, v15.6.05) using the same number of monitor units and identical field setup using Eclipse treatment planning software (v.15.6, Varian Medical Systems, Palo Alto, USA). In cohort 1, one patient had a missing treatment plan due to a cancelled treatment course after the MR imaging and was excluded from body volume and dose analysis but included in the HU assessment. The same dose evaluation was performed for cohort 2, where data was copied from sCT_Acc_DL_1nex to sCT_Acc_orig_2nex.

Gamma evaluation using the dose distributions from sCT_orig and sCT_DL was performed for 22 patients in cohort 1 using MICE toolkit (v.2022.4.9, NONPI Medical AB) with global gamma criteria of 1 %/1mm, 2 %/2 mm, 3 %/3 mm, all with dose cutoff thresholds of 0 %, 10 %, 50 % and 90 % of the prescribed dose. One patient out of the 23 could not be evaluated due to software limitations in the evaluation software. However, no relevant dosimetric deviations were detected for the corresponding dose distributions in the treatment planning system for this patient. Corresponding gamma evaluation was performed in cohort 2 using dose distributions from sCT_Acc_DL_1nex and sCT_Acc_orig_2nex.

A two-sided Wilcoxon signed rank test with a confidence level of 5 % was used from the Python SciPy package (v.1.11.1) [18] for the data analysis of the differences in sCT body volume, HU, and dose.

## 3 Results

**Fig. 2:**
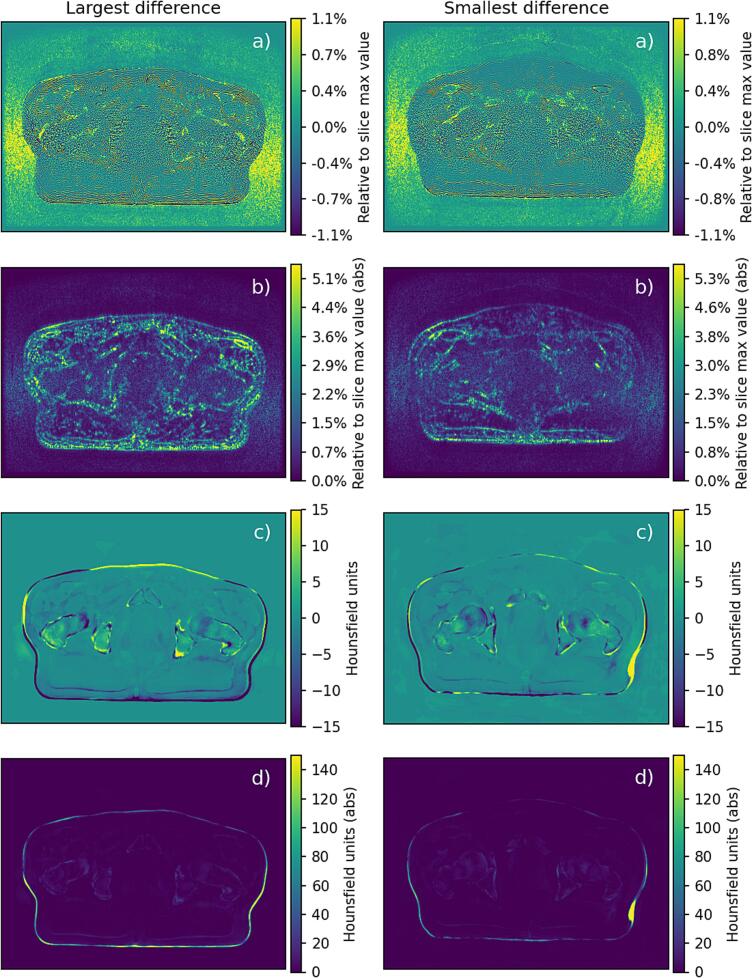
The figures represent differences between MRI using conventional reconstruction and DLR and their corresponding sCT in cohort 1. The left-hand side originates from the patient with the largest absolute value of body volume difference (27.2 cm^3^), and the right-hand side originates from the patient with the smallest absolute value of body volume difference (3.5 cm^3^). The images represent the following: a) Difference image between MR volumes with conventional and DLR reconstruction. Notice the homogeneous content of noise and Gibbs ringing artefacts. Also notice that the image differences do not geometrically correspond to the image differences seen for the sCT. b) Absolute value of image in a) with a wider display window for improved visualization. c) Difference image of the two corresponding sCT volumes for a window width of −15 to 15 Hounsfield units (HU). d) Absolute value of image in c) with a wider window of 0 to 150 HU for improved visualization of peripheral differences. The different window width and level enhance visibility, with higher signal values than the defined scale adopting the color of the highest defined window value. Notice the residual difference in left and right side of the patient for the patient with the largest difference and the small deviation on the patient left side for the patient with the smallest absolute body volume difference (HU scale saturated, true difference value was larger than 150).

All sCTs in both cohorts were of clinically expected image quality determined by visual inspection. The outer contour depicting and encompassing the pelvis resulted in an image volume approximately 0.1 % lower for sCT_orig compared to sCT_DL in cohort 1: median difference −15.7 cm^3^, minimum −27.2 cm^3^ and maximum 7.5 cm^3^ (n = 23), p-value <0.001. The patients with the smallest and largest absolute volume differences measured in the sCT images are provided in Fig. 2.

MAE for every HU interval (sCT_orig-sCT_DL) is reported as the average HU over all subjects in cohort 1 (n = 24) together with ± of the standard deviation (1 SD) and [minimum maximum]: Inside the patient contour 2.9 ± 0.4 [2.3 3.9], outside the patient 1.5 ± 0.4 [1.0 2.7], in fat 1.5 ± 0.3 [1.1 2.3], in muscle 1.2 ± 0.4 [0.8 2.5], in spongy bone 4.9 ± 1.1 [3.8 8.3], and in compact bone 7.4 ± 2.2 [4.5 14.2]. Corresponding data for mean error (ME) in HU was −1.1 ± 0.6 [−2.4 0.2], −0.7 ± 0.4 [−1.4 0.3], −0.5 ± 0.4 [−1.5 0.4], −0.6 ± 0.4 [−2.0 −0.2], 0.8 ± 0.7 [−0.9 2.5], 0.7 ± 1.4 [−3.4 3.9] with p-values <0.001, for all but compact bone where the p-value was 0.01.

**Fig. 3:**
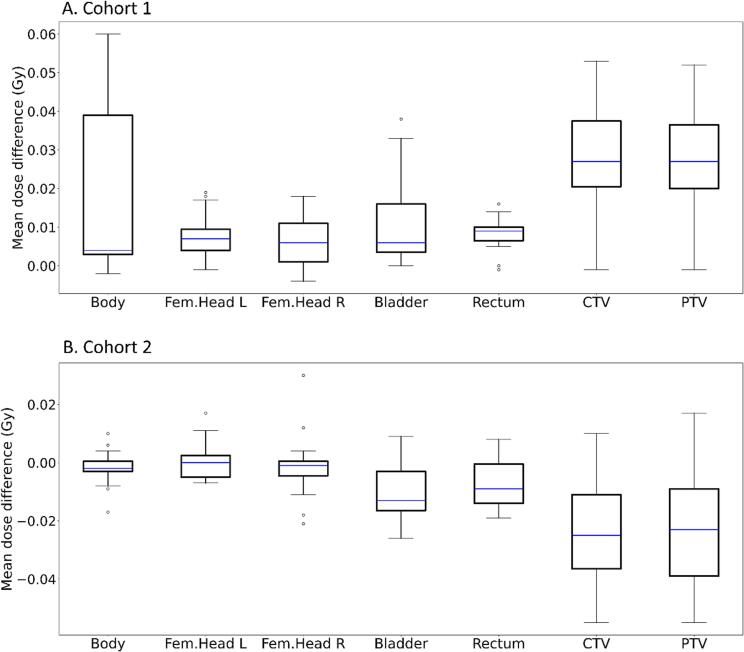
A. Boxplot of mean total dose differences for clinical target volume (CTV) and planning target volume (PTV) and organs at risk (sCT_orig-sCT_DL) in cohort 1 (n = 23). Prescribed dose was 42.7 Gy to planning target volume (PTV). sCT_orig defined the image geometry for the created treatment plan which was copied to sCT_DL and recalculated. B. Boxplot of mean total dose differences for clinical target volume (CTV) and planning target volume (PTV) and organs at risk (sCT_Acc_DL_1nex -sCT_Acc_orig_2nex) in cohort 2 (n = 15). Prescribed dose was 42.7 Gy to planning target volume (PTV). sCT_Acc_DL_1nex defined the image geometry for the created treatment plan which was copied to sCT_Acc_orig_2nex and recalculated.

The mean total dose differences for targets and organs at risks (sCT_orig-sCT_DL) in cohort 1 were all positive and below 0.06 Gy, which corresponds to a 0.1 % difference with respect to the prescribed dose of 42.7 Gy (n = 23, Fig. 3A). Individual p-values were <0.001 for all structures. Corresponding dose difference in cohort 2 (n = 15, sCT_Acc_DL_1nex -sCT_Acc_orig_2nex) also showed differences below 0.06 Gy (n = 15, Fig. 3B). Individual p-values for bladder, rectum, CTV and PTV were 0.004, 0.009, 0.001, and 0.003, respectively and >0.28 for the remaining structures.

The gamma pass rates in cohort 1 were 99.8 % (n = 22) or above for all evaluated dose and distance criteria above 0 % of prescribed dose. Corresponding results for cohort 2 was 99.5 % (Table 1).

**Table 1:** Mean gamma pass rate results in % for distance and dose criteria for multiple dose thresholds in cohort 1 (n = 22) comparing dose calculated on sCT_orig to dose calculated on sCT_DL and cohort 2 (n = 15) comparing dose calculated on sCT_Acc_DL_1nex to dose calculated on sCT_Acc_orig_2nex.

Dose threshold	3 %/3mm global		2 %/2mm global		1 %/1mm global	
Cohort 1	Mean	1 SD	Mean	1 SD	Mean	1 SD
Above 0 % of prescribed dose	100.0	0.0	100.0	0.0	99.8	0.1
Above 10 % of prescribed dose	100.0	0.0	99.9	0.0	99.9	0.1
Above 50 % of prescribed dose	100.0	0.0	100.0	0.0	100.0	0.0
Above 90 % of prescribed dose	100.0	0.0	100.0	0.0	100.0	0.0

Cohort 2	Mean	1 SD	Mean	1 SD	Mean	1 SD

Above 0 % of prescribed dose	100.0	0.0	99.9	0.1	99.5	0.2
Above 10 % of prescribed dose	100.0	0.0	99.9	0.1	99.5	0.3
Above 50 % of prescribed dose	100.0	0.0	100.0	0.0	99.5	0.9
Above 90 % of prescribed dose	100.0	0.0	100.0	0.0	99.9	0.2

## 4 Discussion

DLR is a new method that has been proven to reduce the MR image noise considerably [4]. As a result, in clinical diagnostic radiology the method is implemented with shorter examination times or improved image quality or both [5], [6]. In this study DLR was applied to MR images that were acquired for the purpose of generating sCT using another deep learning-based commercially available method. The combination of these products composed a unique combination of two serially linked commercial deep learning-based products. Our results show that the MR images reconstructed with DLR could be used for sCT generation without sacrificing the sCT integrity. The comparison revealed only minor, clinically negligible, differences in HU and calculated dose. This study also demonstrated a 40 % reduction in MRI acquisition time for sCT generation. This can potentially result in reduced intra-imaging patient motion, hence improved accuracy regarding target delineation and identification of e.g., intra-prostatic fiducial markers.

To the best of our knowledge, this is the first study using deep learning to reconstruct MR images which are subsequently used for deep learning based sCT generation. It is therefore difficult to compare our results to previous studies. The performance of a previous release of MRI Planner has been validated against CT for male pelvis [12], [15], where the overall mean dose differences between sCT and CT dose distributions were below 0.3 %. The corresponding dose differences in this study were below this level (Fig. 3A). Evaluation of sCT images for head and neck treatments using MRI Planner with a similar software version has also been performed with a reported MAE of 67 ± 14 HU within the body contour [19]. The MAE results presented in this study are lower, with the largest MAE of 14.2 HU for compact bone. From recent review papers on deep learning methods for pelvis sCT generation, a MAE between 32 and 51 HU was reported in one paper [20] while an average MAE of 42.4 HU was reported in another review [11]. These errors are larger than ours, however one important aspect must be taken into consideration: In our study, a comparison of results using either sCT or CT is not performed, as the results originate from sCTs generated with and without DLR. The comparison in this study does not rely on different image scan sessions, and it is therefore expected that the differences encountered are smaller than otherwise found in the literature.

The observed MAE and ME in HU and observed dose differences for both cohorts in this study were low and can be considered clinically negligible. However, the dose differences in cohort 1 showed a systematic offset, i.e., the calculated dose was always higher on the original sCT compared to the DL sCT for all targets and organs at risk (Fig. 3A). This was most probably due to the larger outer contour of sCT_DL resulting in a larger body volume compared to sCT_orig. This is supported by the statistically significant results regarding body volume measurement and visually demonstrated in Fig. 2. We therefore have strong evidence to believe that the larger body volume of the sCT_DL (approximately 0.1 %) was due to decreased Gibbs ringing artifacts and reduced MR image noise, due to the DLR. In addition, a change in the body volume could not be visually observed for the MRI volumes, suggesting that it rather was the slightly different image quality around the body-to-air boundary in the MR images that was affecting the sCT generation.

The statistically significant differences in total dose differences for bladder, rectum, CTV and PTV in cohort 2 (Fig. 3B) were probably the result of the structure propagation when RT structures were copied from the clinical image geometry (sCT_Acc_DL_1nex) to the comparing geometry (sCT_Acc_orig_2nex) in combination with patient relaxation and compression during the MR scan session [13]. This relaxation effect has also been observed in previous sCT studies [15].

The investigations performed in our study focused on two effects. The compatibility between two deep learning-based commercial products, i.e. using DLR for MR images as input to a sCT generation software in cohort 1. A strength of this part of the study was that identical MRI k-space data was used in both MR image reconstructions. Thereby, the effects of the DLR method were studied in isolation, with no other confounders. The second effect investigated was the acceleration of the MRI acquisition, enabled by using DLR on data in cohort 2, which allowed for a much shorter scan time with retained MR image SNR and without compromising the sCT quality. The clinical usefulness and value were demonstrated for a revised acquisition protocol and the benefits from using DLR was proven by scan time reduction from 7:00 to 3:51 min (40 % reduction). A limitation of our study was the specific set-up, which does not allow for a generalization of the results to other MRI vendors with other DLR methods.

This study concludes that the integration of two deep learning-based commercial products designed for MRI reconstruction and sCT generation is both compatible and suitable for generating sCT in the pelvic region. Additionally, deep learning-based MRI reconstruction facilitates a reduction in the required MRI scan time for the acquisition sequence intended for sCT generation.

## 5 Availability of data and materials

The present data is summarized in this paper and Supplementary materialsl.

## 6 Ethics approval and consent to participate

The study was approved by the regional ethics board “Regionala Etikprövningsnämnden in Lund”, diary number 2013/742.

## 7 Consent for publication

Not applicable.

## CRediT authorship contribution statement

**Lars E. Olsson:** Conceptualization, Methodology, Investigation, Writing – original draft, Writing – review & editing. **Sacha af Wetterstedt:** Conceptualization, Methodology, Formal analysis, Writing – review & editing. **Jonas Scherman:** Conceptualization, Software, Data curation, Resources, Formal analysis, Writing – review & editing. **Adalsteinn Gunnlaugsson:** Conceptualization, Resources, Writing – review & editing. **Emilia Persson:** Resources, Validation, Investigation, Writing – review & editing. **Christian Jamtheim Gustafsson:** Conceptualization, Software, Visualization, Investigation, Methodology, Formal analysis, Data curation, Funding acquisition, Project administration, Writing – original draft, Writing – review & editing.

## Declaration of competing interest

The authors declare the following financial interests/personal relationships which may be considered as potential competing interests: CJG has received a speaker fee from GE Healthcare. Other authors declare that they have no competing interests.
