# Supplementary material for: Evaluation of a deep learning magnetic resonance imaging reconstruction method for synthetic computed tomography generation in prostate radiotherapy
Source: Phys Imaging Radiat Oncol. 2024 Feb 16;29:100557. doi: 10.1016/j.phro.2024.100557 (PMC10897922; doi:10.1016/j.phro.2024.100557)
Supplement: Supplementary data 1 [file mmc1.docx]

**Supplementary material**


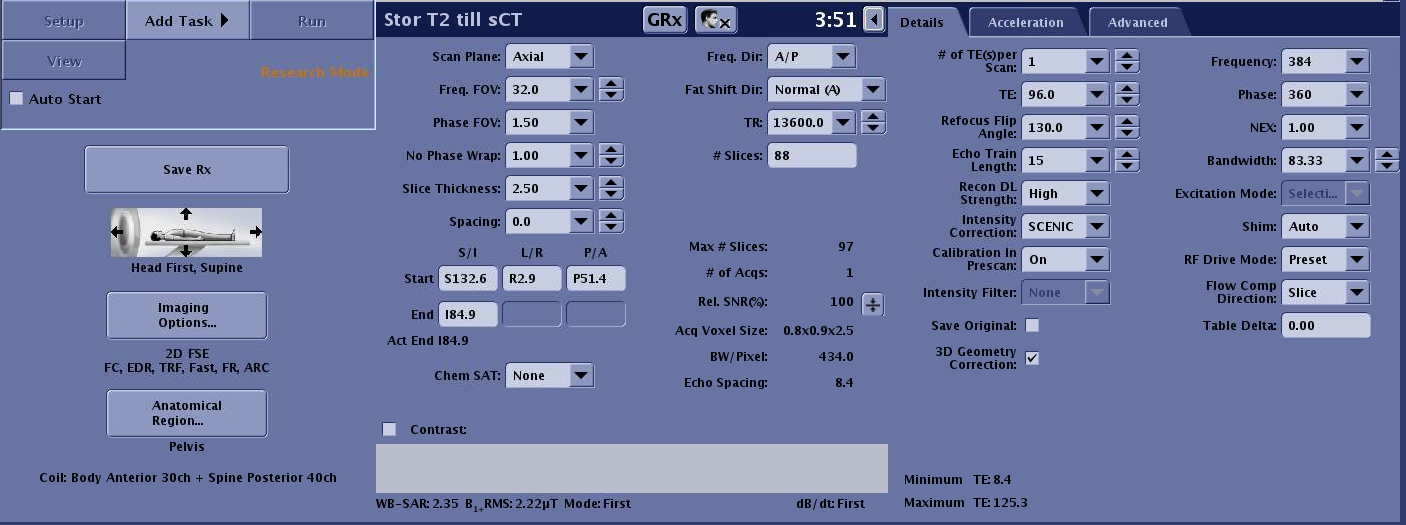


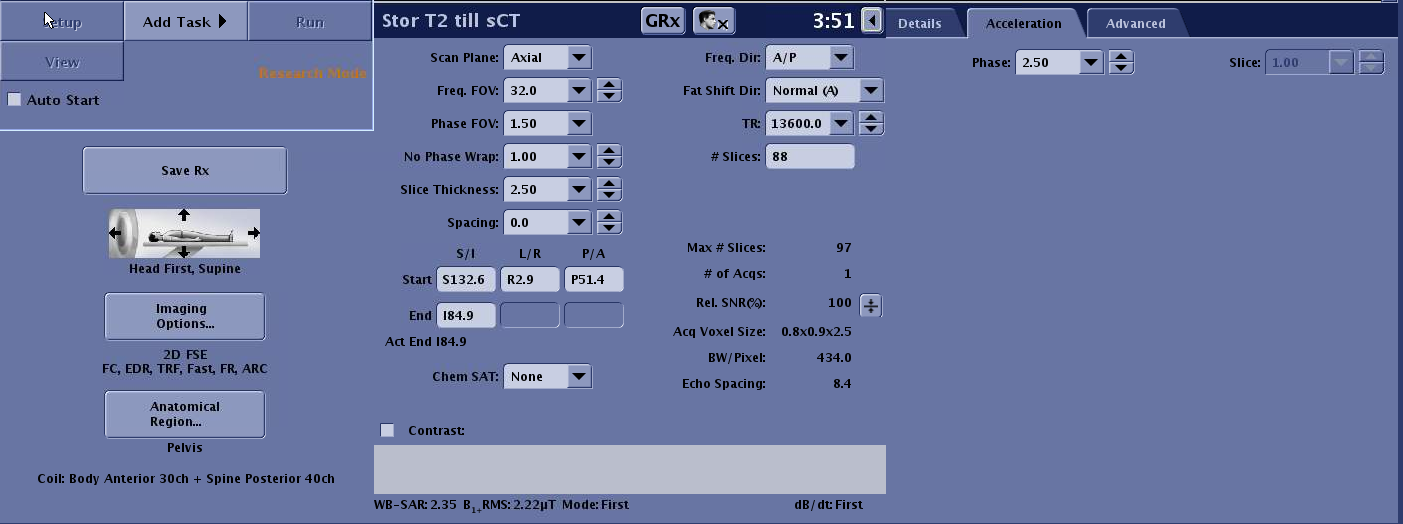


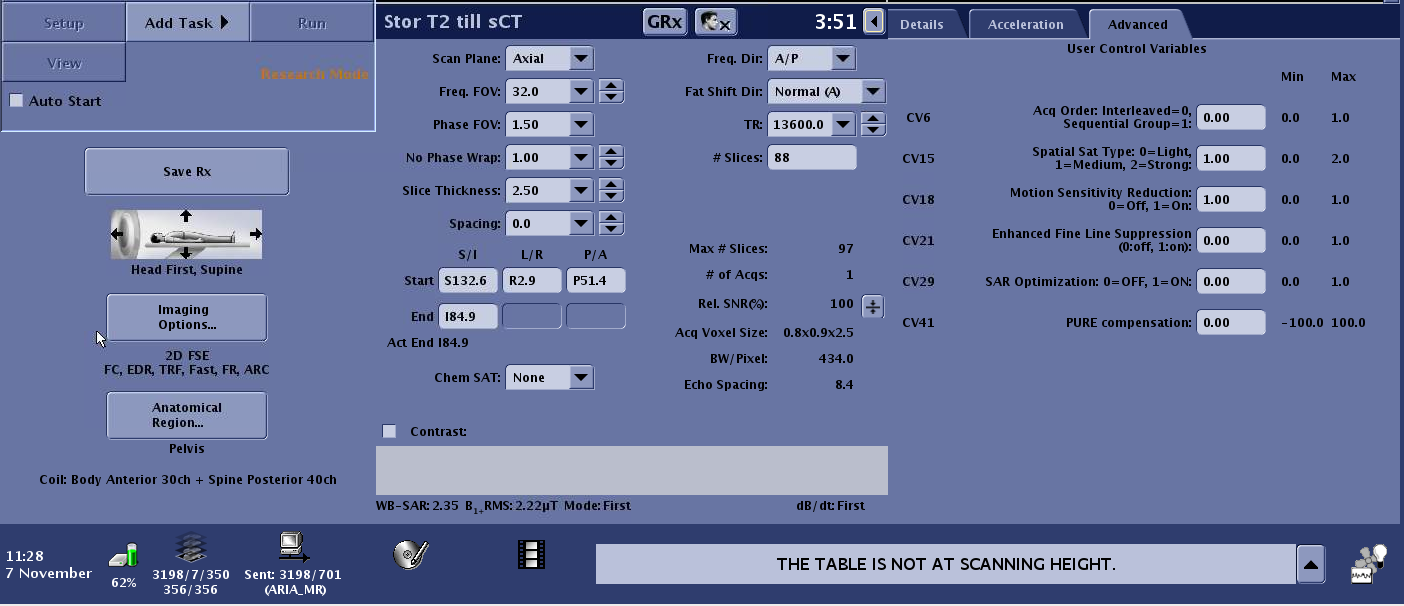


Fig. S1. MRI acquisition protocol for generation of the sCT_Acc_DL_1nex sCT in cohort 2 (Top: Details tab, middle: Acceleration tab and bottom: Advanced tab). This is the clinically used protocol for MRI-Only prostate radiotherapy. Notice the use of mode “High” for the AirReconDL setting (named “Recon DL Strength” in the top figure). By selecting the Recon DL Strength parameter to low, medium, or high the level of signal to noise improvement is determined. By setting it to high it will allow the largest improvement in noise reduction.


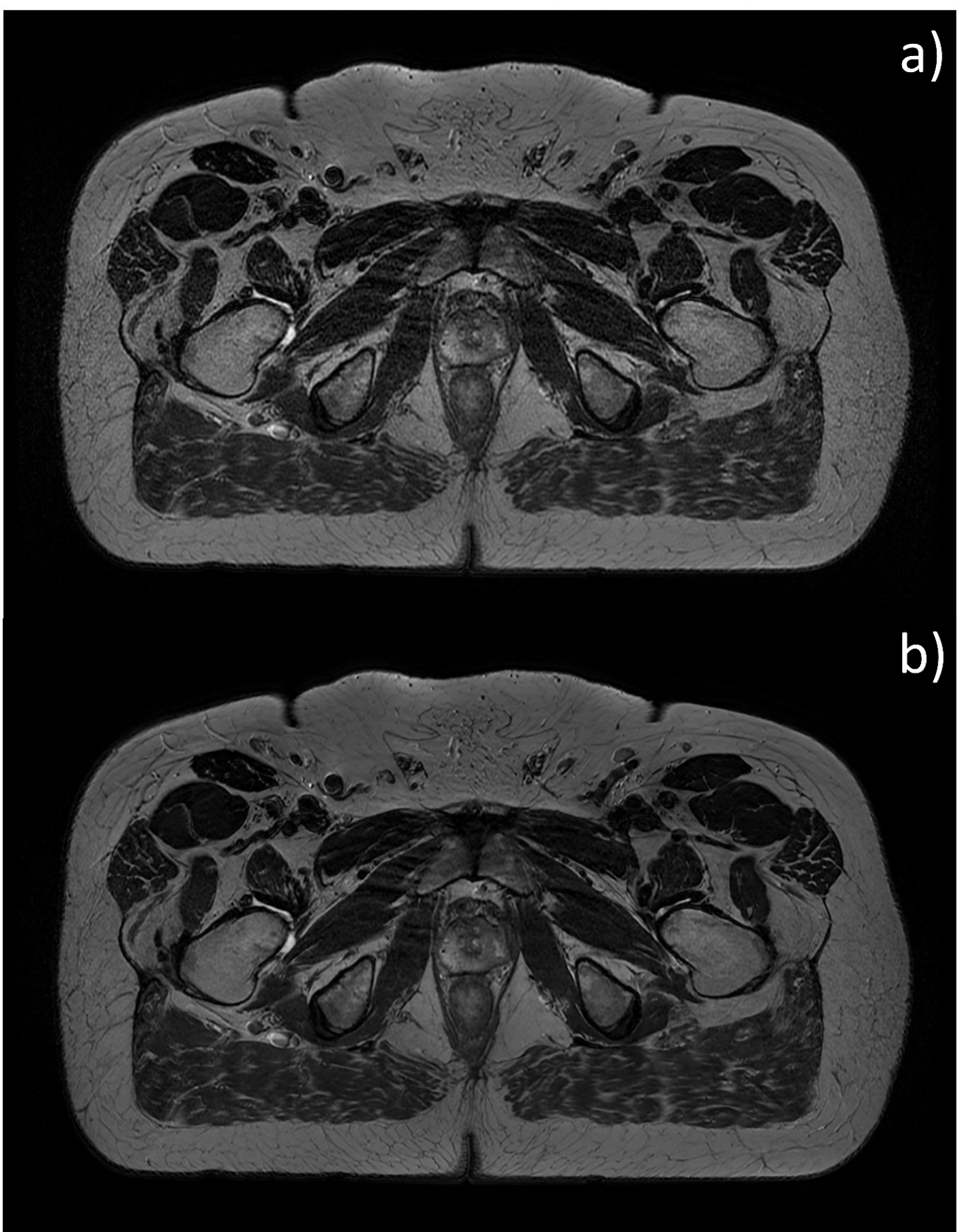


Fig. S2. MR image slice of the patient with the largest body volume in cohort 1, scan time 7:00 minutes. Reconstructed with a) conventional MRI image reconstruction, b) Air Recon DL MR image reconstruction. Notice the decreased image noise level and Gibbs ringing compared to a). Corresponding sCT images for a) and b) can be seen in Fig. S5a and Fig. S5b, respectively.


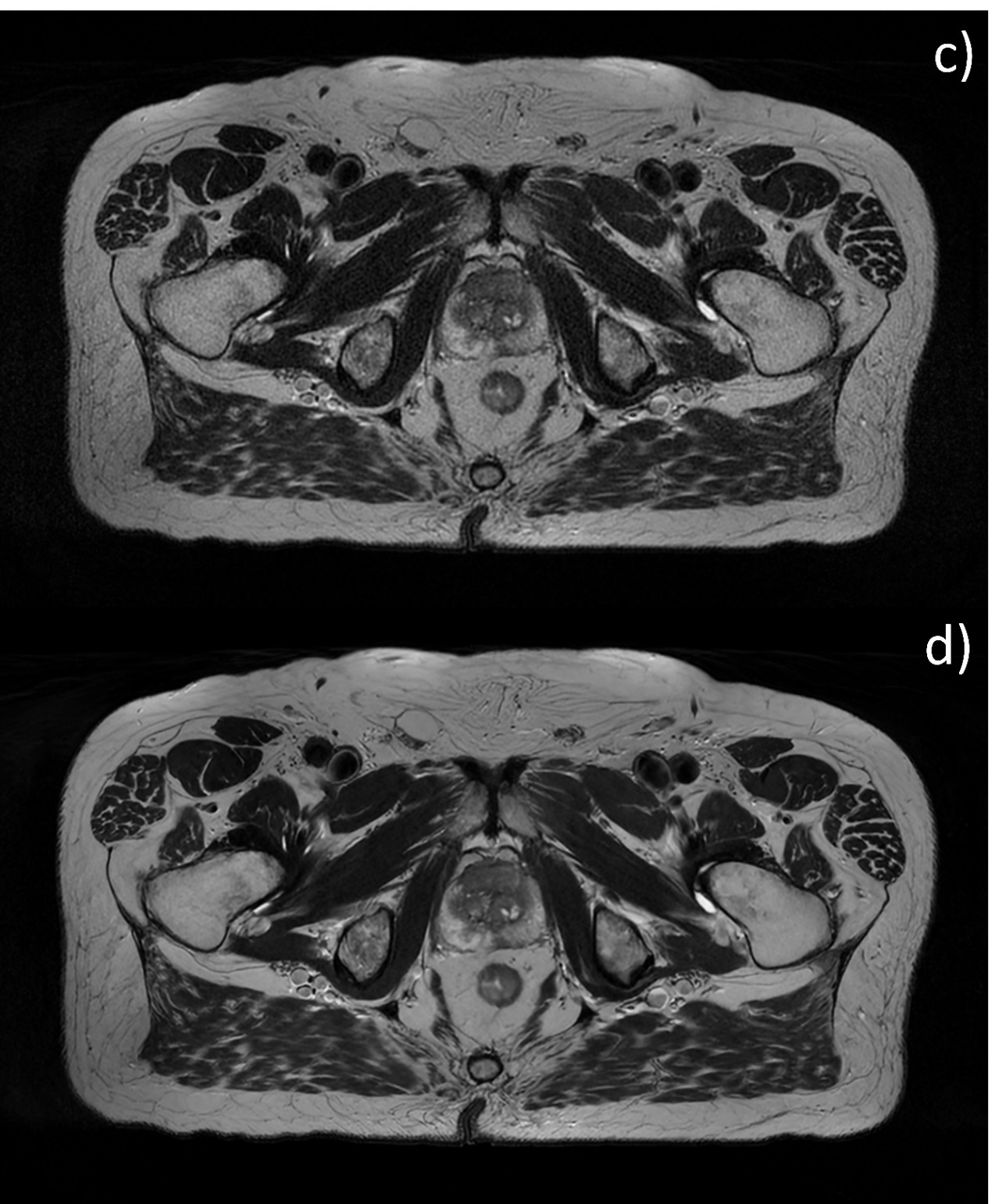


Fig. S3. An MR image slice of the patient with the largest body volume in cohort 2 scanned with the default clinical sequence, scan time 3:51 minutes, see Fig. S1 for acquisition protocol. Reconstructed with c) conventional MR image reconstruction (this data was not used in the study, only presented here to demonstrate the capabilities of Air Recon DL), d) Air Recon DL MR image reconstruction (used by default in clinic), corresponding sCT image can be seen in Fig. S6d. The acqusition protocol was optimized to use Air Recon DL. Notice the decreased image noise level and decreased Gibbs ringing in d) compared to c). Also, notice the similar image quality in d) compared to e) in Fig. S4 which was scanned with a double scan time, but reconstructed with conventional image reconstruction.


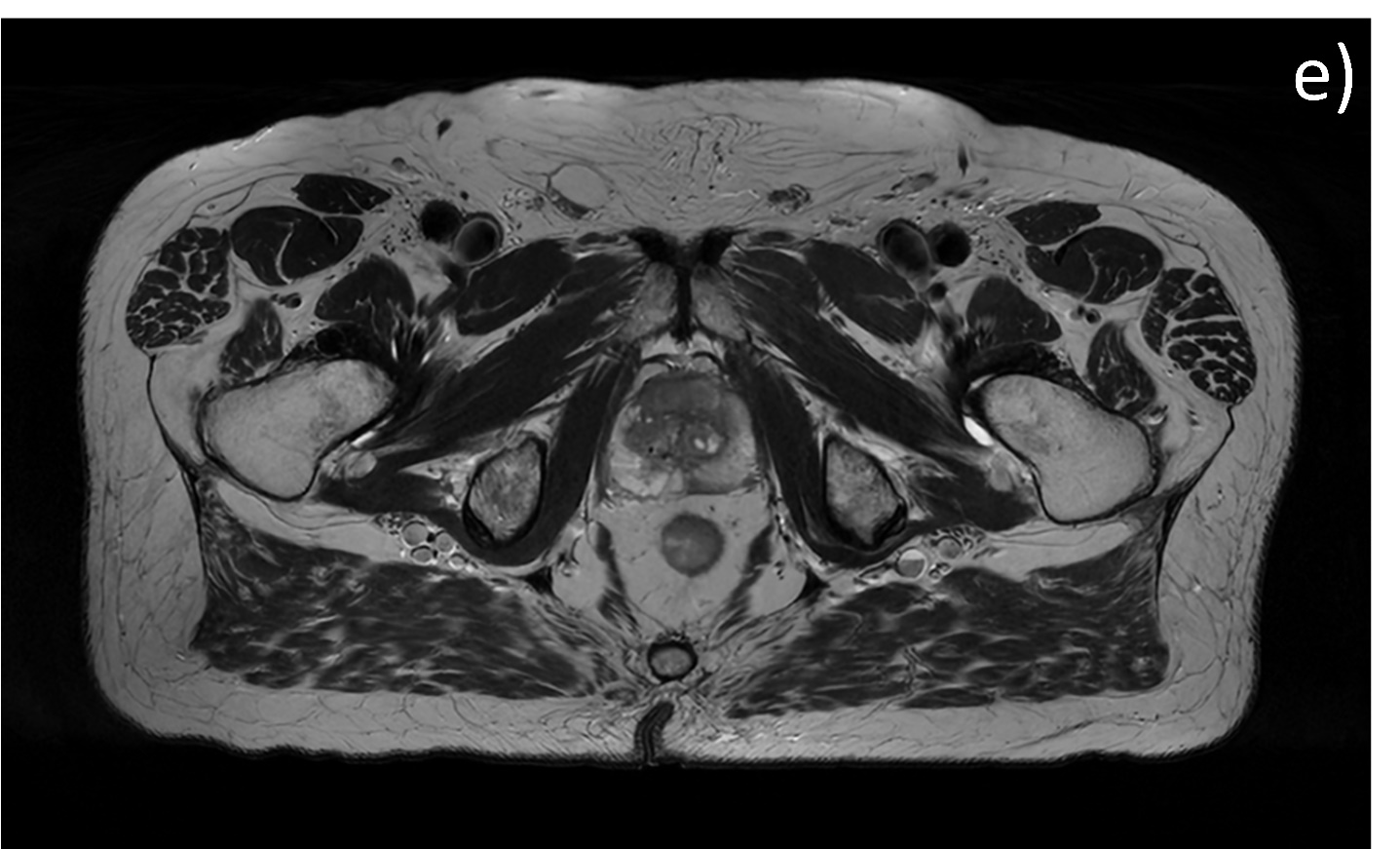


Fig. S4. The same image slice position as in Fig. S3 of the patient with the largest body volume in cohort 2 scanned with the clinical sequence but the number of acqusition averages was set to two, scan time 7:29 minutes. Corresponding sCT image can be seen in Fig. S6e. Reconstructed with conventional image reconstruction. A similar image quaility could be reached in half the scan time when reconstructed with Air Recon DL, see Fig. S3d.


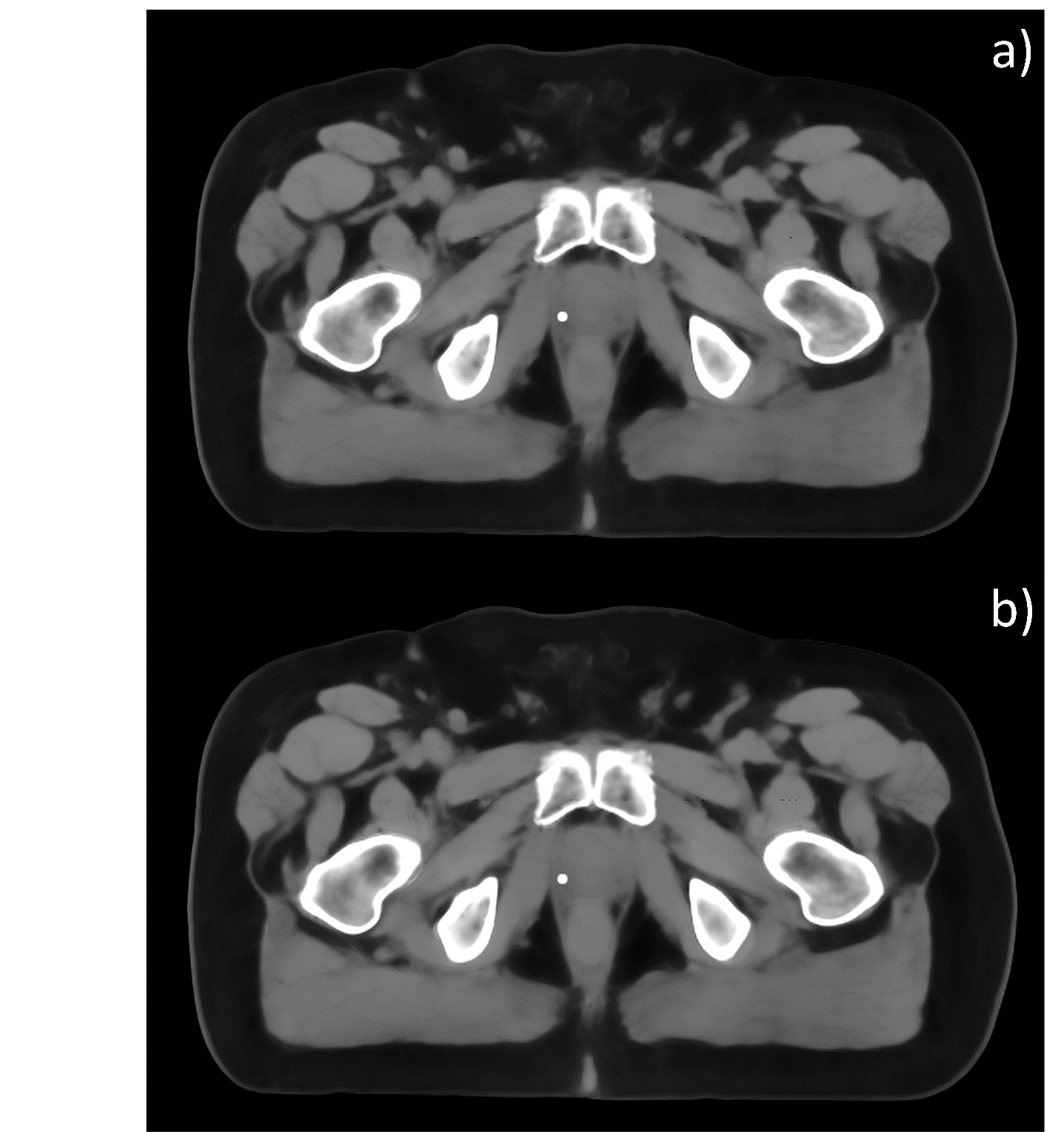


Fig. S5. sCT image slice of the patient with the largest body volume in cohort 1. Source MR image slice reconstructed with a) conventional MRI image reconstruction, b) Air Recon DL MR image reconstruction. Notice the similar sCT image appearance. Corresponding MR images for a) and b) can be seen in Fig. S2a and Fig. S2b, respectively .


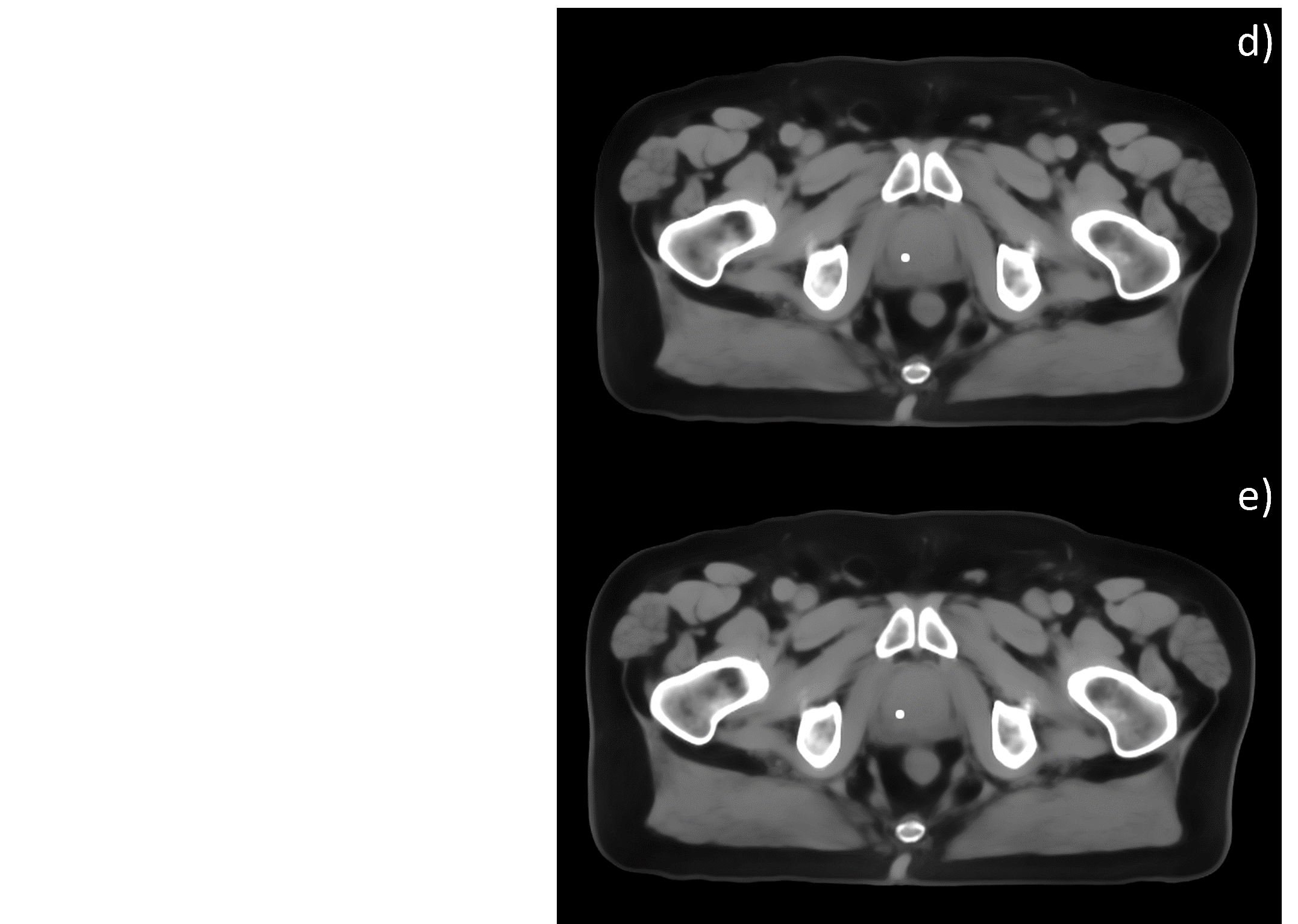


Fig. S6. sCT image slice of the patient with the largest body volume in cohort 2. Source MR image slice reconstructed with a) Air Recon DL MRI image reconstruction, b) conventional MR image reconstruction. Notice the similar sCT image appearance. Corresponding MR images for d) and e) can be seen in Fig. S3d and Fig. S4e, respectively.
